# Osteocyte‐Like Cells Differentiated From Primary Osteoblasts in an Artificial Human Bone Tissue Model

**DOI:** 10.1002/jbm4.10792

**Published:** 2023-06-28

**Authors:** Arooj Munir, Janne Elin Reseland, Hanna Tiainen, Håvard Jostein Haugen, Pawel Sikorski, Emil Frang Christiansen, Finn Per Reinholt, Unni Syversen, Lene Bergendal Solberg

**Affiliations:** ^1^ Department of Biomaterials Institute of Clinical Dentistry, University of Oslo Oslo Norway; ^2^ Department of Physics Norwegian University of Science and Technology (NTNU) Trondheim Norway; ^3^ Department of Pathology Oslo University Hospital Oslo Norway; ^4^ Department of Clinical and Molecular Medicine Norwegian University of Science and Technology Trondheim Norway; ^5^ Division of Orthopedic Surgery Oslo University Hospital Oslo Norway

**Keywords:** ARTIFICIAL BONE TISSUE, ORGANOIDS, OSTEOBLASTS, OSTEOCYTES, SPHEROIDS

## Abstract

In vitro models of primary human osteocytes embedded in natural mineralized matrix without artificial scaffolds are lacking. We have established cell culture conditions that favored the natural 3D orientation of the bone cells and stimulated the cascade of signaling needed for primary human osteoblasts to differentiate into osteocytes with the characteristically phenotypical dendritic network between cells. Primary human osteoblasts cultured in a 3D rotating bioreactor and incubated with a combination of vitamins A, C, and D for up to 21 days produced osteospheres resembling native bone. Osteocyte‐like cells were identified as entrapped, stellate‐shaped cells interconnected through canaliculi embedded in a structured, mineralized, collagen matrix. These cells expressed late osteoblast and osteocyte markers such as osteocalcin (OCN), podoplanin (E11), dentin matrix acidic phosphoprotein 1 (DMP1), and sclerostin (SOST). Organized collagen fibrils, observed associated with the cell hydroxyapatite (HAp) crystals, were found throughout the spheroid and in between the collagen fibrils. In addition to osteocyte‐like cells, the spheroids consisted of osteoblasts at various differentiation stages surrounded by a rim of cells resembling lining cells. This resemblance to native bone indicates a model system with potential for studying osteocyte‐like cell differentiation, cross‐talk between bone cells, and the mineralization process in a bonelike structure in vitro without artificial scaffolds. In addition, natural extracellular matrix may allow for the study of tissue‐specific biochemical, biophysical, and mechanical properties. © 2023 The Authors. *JBMR Plus* published by Wiley Periodicals LLC on behalf of American Society for Bone and Mineral Research.

## Graphical abstract

Graphical abstract showing culture of human primary osteoblast in a material‐free 3D rotating bioreactor with combinations of vitamins for up to 21 days, resulting in the formation of a single spheroid, referred to as an osteospheroid. The osteopheroid showed bonelike structures with entrapped stellate‐shaped cells interconnected with canaliculi embedded in mineralized collagen matrix, typical of osteocytes.

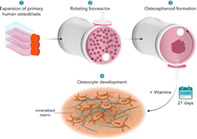

## Introduction

Osteocytes, the most abundant cell type of bone, are the key regulators of bone formation and resorption. They also function as endocrine cells producing hormones that integrate skeletal metabolism and global mineral and nutrient homeostasis.^(^ ^1^, ^2^ ^)^ The investigation of osteocytes is challenging, as the access to osteocytes and their network in vivo and ex vivo is hampered by their deeply embedded location in mineralized tissue. It is difficult to isolate the osteocytes from their three‐dimensional (3D) native niche and maintain their phenotype and function under two‐dimensional (2D) culturing conditions, as the natural 3D osteocyte orientation is required to develop a dendritic network for their communication and function.^(^ ^3^ ^)^

Traditionally, conventional 2D culturing conditions have been viewed as a gold standard to explore the underlying cellular mechanisms of bone remodeling.^(^ ^4^ ^)^ However, the 3D culturing environment has several advantages over 2D, such as allowing the cells to grow and interact with the surrounding cells and the extracellular matrix secreted by the cells without disruption by trypsination.^(^ ^5^ ^)^ 3D models consisting of natural and/or synthetic scaffolds, for example, collagen, cadaveric bone, bone chip, calcium phosphate particles, polymers, and ceramic^(^ ^6^, ^7^, ^8^, ^9^, ^10^ ^)^ scaffolds, have been applied to investigate bone cell growth. Microbeads have been used to mimic the lacuna canaliculi network of osteocytes, to facilitate the differentiation of osteoblasts to osteocyte, osteocyte gene expression, and to reproduce the phenotype of terminally differentiated osteocytes.^(^ ^11^, ^12^, ^13^ ^)^ Such artificial bone structures provide a 3D environment to cells, but they may fail to mimic the natural in vivo cellular events that occur during osteoblast to osteocyte differentiation, and the material(s) used may interact with the bone formation.^(^ ^4^ ^)^ 3D rotary cell cultures can be used to study osteocyte differentiation and communication ex vivo. These culturing conditions may not only simulate microgravity but also provide the dynamic culturing system that facilitates co‐localization, aggregation, and differentiation of cells,^(^ ^14^ ^)^ cell‐to‐cell, and cell‐to‐extracellular matrix interaction,^(^ ^15^ ^)^ hence replicating the 3D architecture of native tissue. Moreover, the production of extracellular matrix has a better similarity to the in vivo situation when compared with other models.^(^ ^16^ ^)^ 3D rotary cultures have been applied to study cell aggregation,^(^ ^17^ ^)^ osteoclastogenesis with^(^ ^18^ ^)^ or without^(^ ^19^ ^)^ a scaffold, as well as osteoblastogenesis.^(^ ^20^, ^21^ ^)^ Studies have shown that 3D rotary culture enhances the osteogenic potential of human bone marrow stromal cells^(^ ^22^ ^)^ and human amniotic fluid mesenchymal stem cells^(^ ^23^ ^)^ and the mineralization of constructs from primary human osteoblasts.^(^ ^24^ ^)^ Additionally, the effect of adiponectin,^(^ ^25^ ^)^ parathyroid hormone (PTH),^(^ ^26^ ^)^ vitamin K, and vitamin D3^(^ ^24^ ^)^ on the osteogenic potential and the mechanical properties of bonelike constructs generated from primary cells and cell lines have been investigated in 3D rotary cell systems.

In addition to optimal culturing conditions, with a constant supply of nutrients and minerals, vitamins may also play a role in the osteocyte differentiation ex vivo. Ascorbic acid (AA, or vitamin C) is involved in proliferation and differentiation of bone cells and mesenchymal cells^(^ ^27^, ^28^ ^)^ and is used as an important supplement for maintenance of the osteoblast phenotype and/or osteogenic differentiation in vitro.^(^ ^29^ ^)^ Vitamin D is well known to regulate calcium and phosphate homeostasis and induces proliferation and maturation of osteoblasts and osteocytes and increases bone markers such as receptor activator of NF‐κB ligand/osteoprotegerin (RANKL/OPG) and fibroblast growth factor 23 (FGF23).^(^ ^30^, ^31^ ^)^ Retinoic acid (RA, a metabolite of vitamin A) is responsible for various cellular functions, such as cell proliferation and differentiation,^(^ ^32^ ^)^ and studies have shown that RA reduces osteoblast proliferation and induces osteoblast to osteocyte differentiation.^(^ ^33^ ^)^ The ideal culturing conditions mimicking the natural environment necessary to establish a model for human osteocytes in vitro have yet to be found. As osteocytes require the presence of 3D extracellular mineralized matrix conditions in which to differentiate and function,^(^ ^34^ ^)^ we hypothesize that a 3D rotary culturing system in addition to a selection of vitamins will generate osteocytes from human osteoblasts in a scaffold‐free environment. Consequently, primary human osteoblasts were cultured in a scaffold‐free rotary environment together with selected vitamins generating osteospheroids. Different histological and microscopical techniques were used to examine the spheroids that revealed bonelike structures with organized and mineralized extracellular matrix, entrapped cells with osteocytic appearance, as well as cells with positive stain for specific osteocyte markers.

## Materials and Methods

### Cell culture

Commercially available primary human osteoblasts (hOBs) from two different adult donors were obtained from Lonza (Walkerville, MD, USA). The cells were cultured in osteoblast growth medium (PromoCell GmbH, Heidelberg, Germany) supplemented with SupplementMix (PromoCell GmbH) and 1% penicillin and streptomycin (PAA Laboratories GmbH, Pasching, Austria). The cells were incubated at 37°C and 5% CO_2_ until 80% to 90% confluency. The medium was changed every third day.

### Three‐dimensional cell culture

The spheroids were generated in the absence of any foreign material in a rotary culture. Briefly, 3–4 × 10^6^ hOBs were transferred to each disposable CelVivo bioreactor vessel (10 mL) (cat. no. DM 010; CelVivo, Blommenslyst, Denmark) and incubated in a humidified BioArray Matrix drive BAM v4 (CelVivo) at 37°C and 5% CO_2_. The cells were supplemented with osteogenic differentiation medium (200 nM hydrocortisone [HC], 10 mM β‐glycerophosphate [βGP], and 50 μM ascorbic acid [AA]; Sigma‐Aldrich, St. Louis, MO, USA) as well as all‐trans‐retinoic‐acid (ATRA) (10 μM) (Sigma‐Aldrich) and vitamin D (25(OH) D3) (10^−6^ M) (Calcifediol CRS; European Pharmacopoeia Reference Standard, EDQM, Strasbourg, France). To facilitate osteoblast aggregation and osteospheroid formation, the initial speed of the bioreactors was kept at 1 rpm for 2 days, allowing cells to aggregate in one spheroid, and on day 3 the rotation speed of the BAM was enhanced to 4 rpm until the end of the experiment. The osteogenic differentiation medium with freshly added vitamins was changed twice a week. Osteospheroids were harvested on day 14 (D14) and day 21 (D21) and were approximately 1.5 to 2 mm in diameter. The spheroids harvested were cut in two with a scalpel; one half was fixed overnight in 4% formalin (F) for histology, and the second half was fixed overnight in 2% glutaraldehyde (GA) for electron microscopy.

Spheroids were harvested in two independent experiments using same settings with commercially available hOBs (Lonza) from two different adult donors. In addition, a spheroid harvested from the third experiment was used for cryo‐embedding and the histological data are shown in Supplemental Figure S2. Furthermore, the data obtained are not presented as pooled.

### Histological tissue preparation

The spheroids were embedded in paraffin and serial sections of 3‐ to 4‐μm thickness were cut using a microtome (HM 355 S, Thermo Fisher Scientific, Waltham, MA, USA). After deparaffinization in xylene and rehydration through graded series of ethanol (100% to 70%), one set of sections was stained according to Masson‐Goldner for the study of morphological features, and in the following sections, selected osteocyte markers were identified by immunohistochemistry.

### Masson‐Goldner staining

Nuclei were stained for 5 minutes with Weigart's hematoxylin (Chemi‐teknik AS, Oslo, Norway), followed by rinsing with acid alcohol and water. Subsequently, the tissue components were stained with ponceau‐syrefuchsin‐azophloxin (Fuchsin acid: MERCK, Darmstadt, Germany; Ponceau: Chemi‐teknik AS, Oslo, Norway) for 7 minutes, orange G (Sigma‐Aldrich) for 5 minutes, and 0.5% light green (Chemi‐teknik AS) for 7 minutes, and in‐between washing with 1% acetic acid was performed. Finally, sections were rinsed in acetic acid, dehydrated quickly through graded ethanol and xylene, and mounted. The mounted sections were analyzed using light microscopy (Olympus BX51 microscope, Tokyo, Japan).

### Immunohistochemistry

Antigen retrieval was carried out by incubating the sections with hyaluronidase (15.000 U/mL, Sigma‐Aldrich) in a hot cabinet at 37°C for 30 minutes, followed by EnVision FLEX Wash Buffer 1× (Dako‐Agilent, Santa Clara, CA, USA). Sections were blocked with endogenous peroxidase (Dako‐Agilent) for 5 minutes and 1% BSA (Sigma‐Aldrich) for 30 minutes, both at room temperature (RT), before incubation with the respective primary antibodies: rabbit dentin matrix acidic phosphoprotein 1 (DMP1) (1:200; HPA037465; Sigma‐Aldrich), rabbit sclerostin (1:50; ab85799; Abcam, Cambridge, UK), sheep Podoplanin (E11) (1:50; AF3670; R&D Systems, Minneapolis, MN, USA), and mouse osteocalcin (OCN) (1:00; 33–5400; Thermo Fisher Scientific) overnight at 4°C. Incubation with appropriate secondary antibodies envision + system‐HRP anti‐rabbit (Dako‐Agilent), anti‐mouse (Dako‐Agilent), and IgG HRP‐conjugated anti‐sheep (1:500; R&D Systems) were performed for 30 minutes at RT followed by washing. Finally, the sections were stained with hematoxylin, dehydrated, and mounted. For the negative control, sections were stained without primary antibodies. The mounted sections were analyzed using an Olympus BX51 microscope.

### Transmission electron microscopy (TEM) sample preparation

After the glutaraldehyde (GA) fixation, samples were embedded in an epoxy resin, and ultra‐thin sections (75 nm) were cut with an ultramicrotome (RM2265, Leica, Microsystems, Wetzlar, Germany). The sections were mounted on formvar‐coated nickel slot grids and analyzed under conventional TEM (Tecnai 12, FEI Company, Eindhoven, the Netherlands).

### Scanning electron diffraction (SED)

The scanning electron nanobeam diffraction data were acquired on a field emission transmission electron microscope (JEM2100F, JEOL, Tokyo, Japan) operated at 200 kV. A probe with nominal spot size 0.5 nm was raster scanned over the sample using an external scan generator (P1000, NanoMEGAS, Brussels, Belgium), and a hybrid pixel direct electron detector (MerlinEM 1S, Quantum Detectors, Harwell Oxford, UK) with 256 × 256 pixels was used to acquire diffraction patterns with an exposure time of 10 ms. Packages HyperSpy v1.5.2 (https://hyperspy.org) and pyxem v0.12 (https://github.com/pyxem/pyxem) were used in data processing.^(^ ^35^, ^36^ ^)^

### Scanning electron microscopy‐energy dispersive X‐ray spectroscopy (SEM‐EDX)

Paraffin‐embedded sections of 3‐ to 4‐μm thickness from day 14 spheroid were mounted on glass slides and then on the aluminum stub with conductive carbon tape. In addition, sample surface was connected to the aluminum sample holder using conductive copper tape to reduce charging. The surface morphology was visualized using a scanning electron microscope (SEM; TM3030; Hitachi, Japan) equipped with a detector energy‐dispersive X‐ray spectroscope (EDX) (Quantax70; Hitachi, Japan). Imaging was performed using backscattered electrons in charge reduction mode with 15 kV acceleration voltage. SEM allowed the identification of structural areas of interest in the osteospheroid, and EDX was used to determine the elemental composition in the areas of interest.

## Results

### Confirmation of the bonelike structure in the osteospheroids

**Fig. 1:**
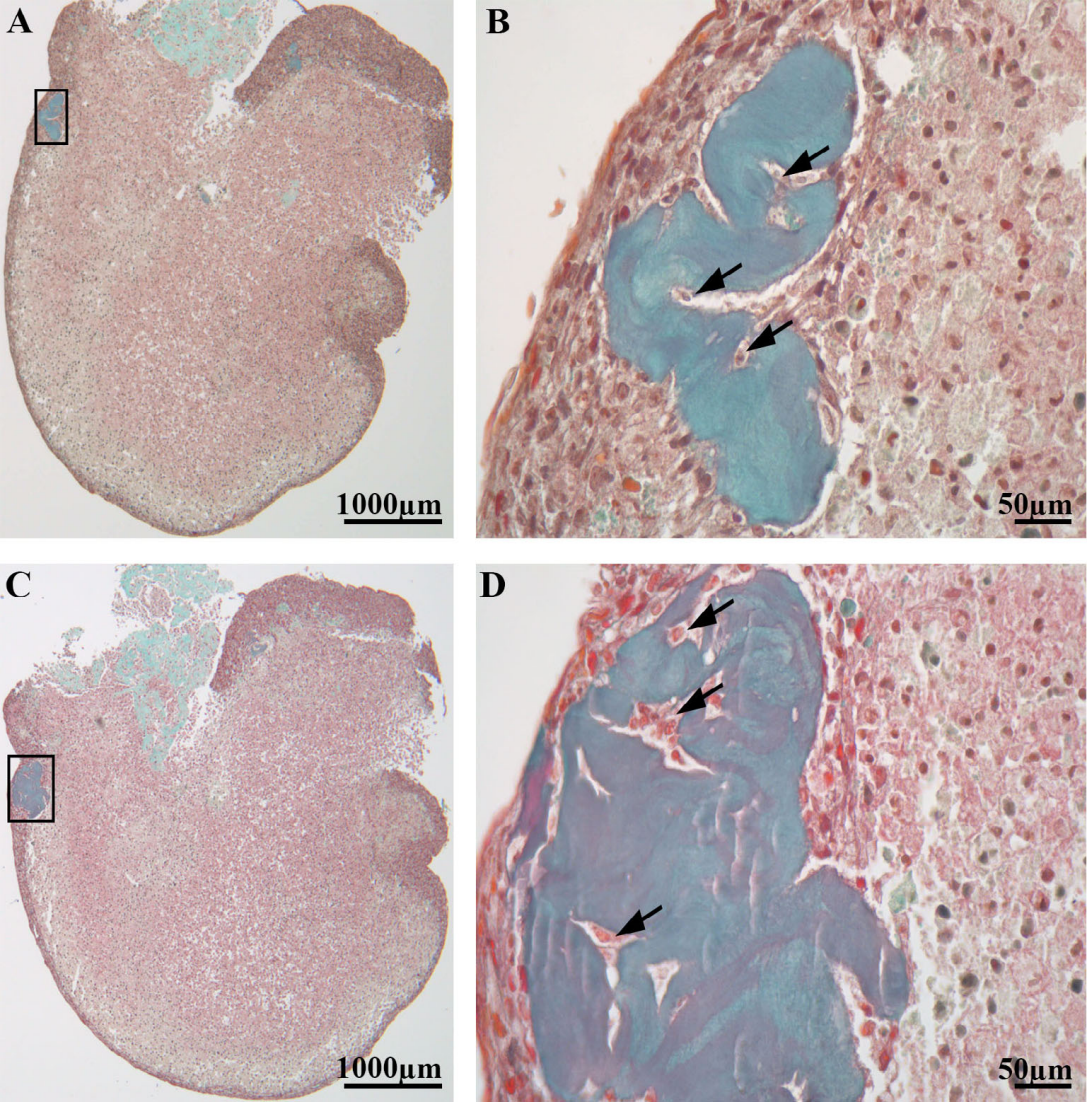
Osteospheroid harvested at day 14 sectioned at different layers. (*A*, *C*) Representative images of two of the collagen‐rich areas within the same osteospheroid. The spheroid displays an outer rim of densely packed cells. Within the osteopheroid, the cells are more loosely packed with areas of collagen (green, arrows in *A* and *C*) mainly in association with the outer rim. These collagen‐rich areas also display entrapped cells. Higher magnification of the black rectangular areas from *A* and *C* are visualized on *B* and *D*, showing the entrapped cells (black arrows) within the collagen‐rich areas.

Histological staining was carried out to study the cross‐sectional morphology and distribution of collagen and bone cells in the osteospheroids. Staining with Masson‐Goldner trichrome (Fig. 1) showed that the cells along the periphery of the osteospheroid were closely packed and formed an outer rim of the osteospheroid compared with the cells within the osteospheroid, which were loosely arranged. Several collagen‐rich areas were also observed close to the outer rim of the osteospheroids at day 14 (D14) (Fig. 1). These areas were characterized by entrapped cells surrounded by a collagenous matrix. The collagen matrix was produced by the cells in the osteospheroid and some cells were most likely entrapped in their own matrix during the process, resulting in these bonelike structures (Fig. 1*B*, *D*). Similar location, shape, and size of the bonelike structures were observed in osteospheroids at day 21 (D21) (results not shown).

**Fig. 2:**
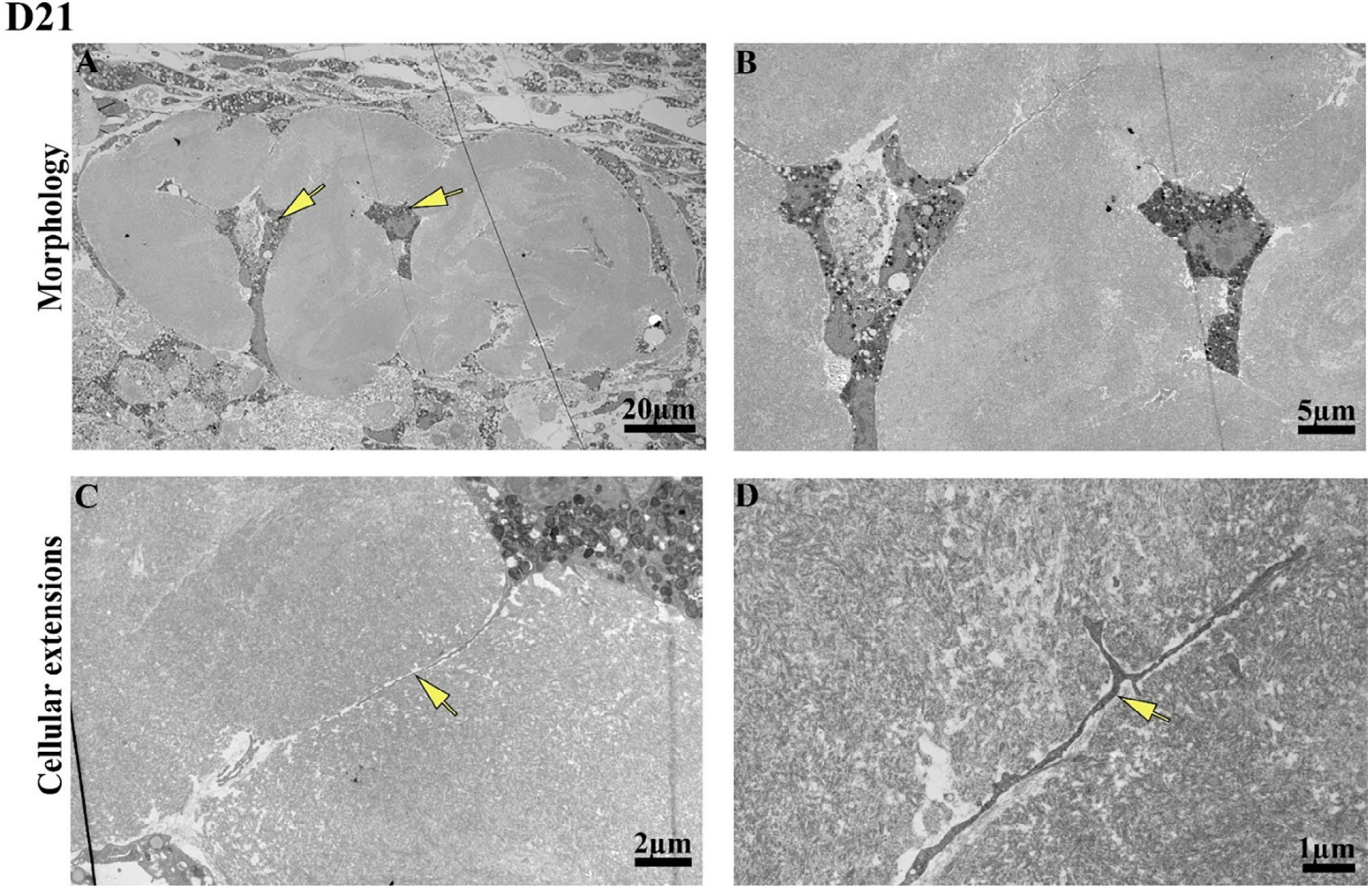
Micrographs from transmission electron microscopy (TEM) analyses at day 21 (D21). (*A*) TEM image of an osteospheroid at D21 with entrapped cells (arrows) surrounded by dense collagen in a bonelike structure. (*B*) The entrapped cells in *A* at a higher magnification. (*C*, *D*) The cellular extensions identified in the bonelike structure.

**Fig. 3:**
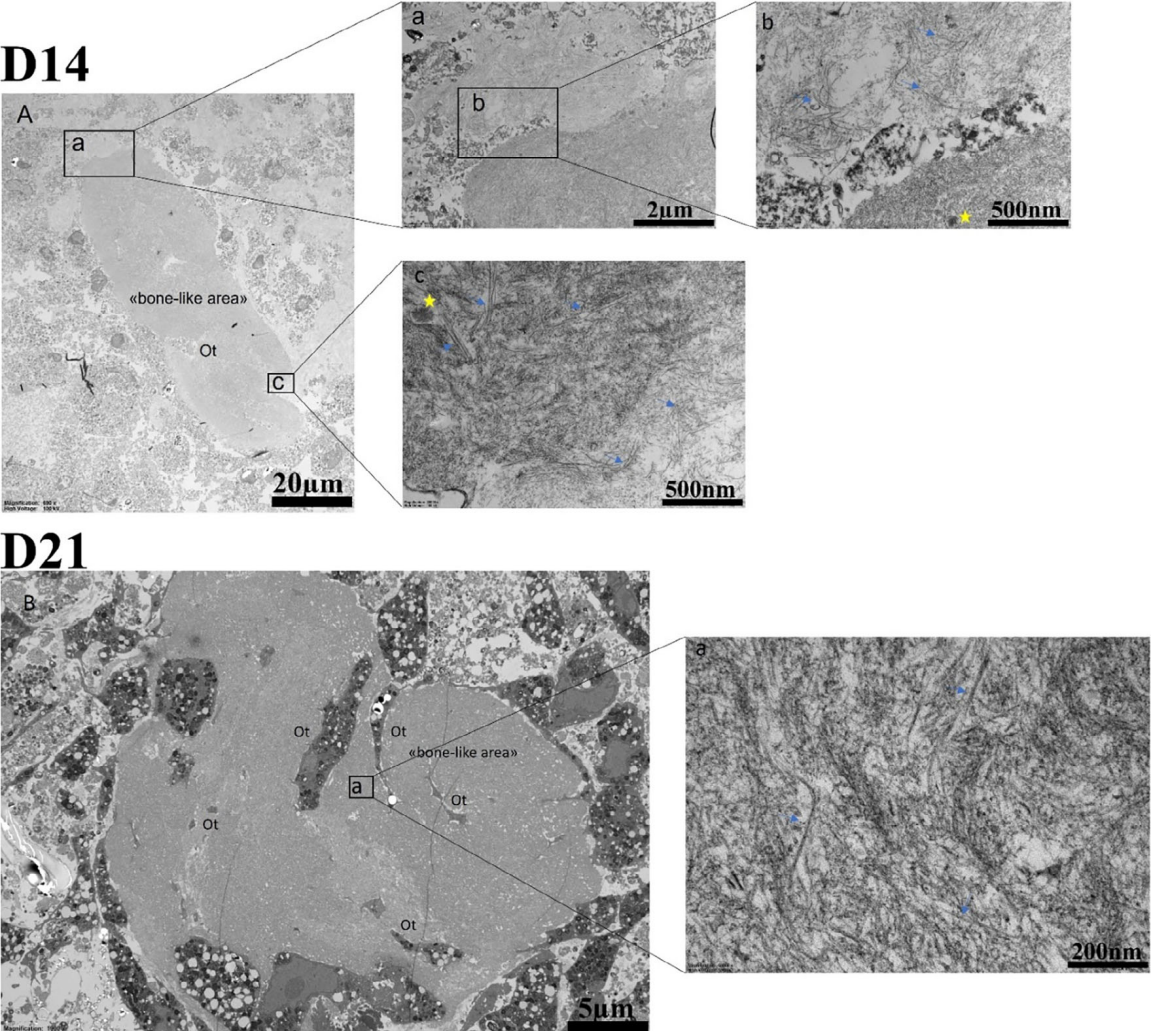
Micrographs from transmission electron microscopy (TEM) analysis at day 14 (D14) and day 21 (D21). (*A*) Overview of a bonelike area in the osteospheroid on D14. (*Aa*) The interphase between loose and dense collagen. (*Ab*, *Ac*) The collagen fibrils (arrows). There are also crystals in between the collagen fibrils (yellow stars). (*B*) TEM section of bonelike area in osteospheroid on D21. (*Ba*) The collagen fibrils (arrows) surrounding and embedding the cells.

The histological findings were supported by TEM analyses of the osteospheroids at D21 (Fig. 2). TEM images of the osteospheroids showed a bonelike structure with deeply embedded cells The embedded cells had a stellate shape (Fig. 2*B*) and with cellular extensions running within the bonelike structure and toward other cells (Fig. 2*C*, *D*). In addition, loose banded collagen fibrils were identified adjacent to the bonelike structure on D14 (Fig. 3*A*). Similarly, dispersed and organized collagen fibrils along with small aggregates of needle‐shaped structures suggestive of mineral crystals (Fig. 3*Ac*) were observed on another location in bonelike structure on D14. At D21, more densely packed collagen fibrils were observed (Fig. 3*B*). However, no crystals were observed within the collagen matrix on D21.

### Detection of crystalline calcium phosphate using scanning electron diffraction

**Fig. 4:**
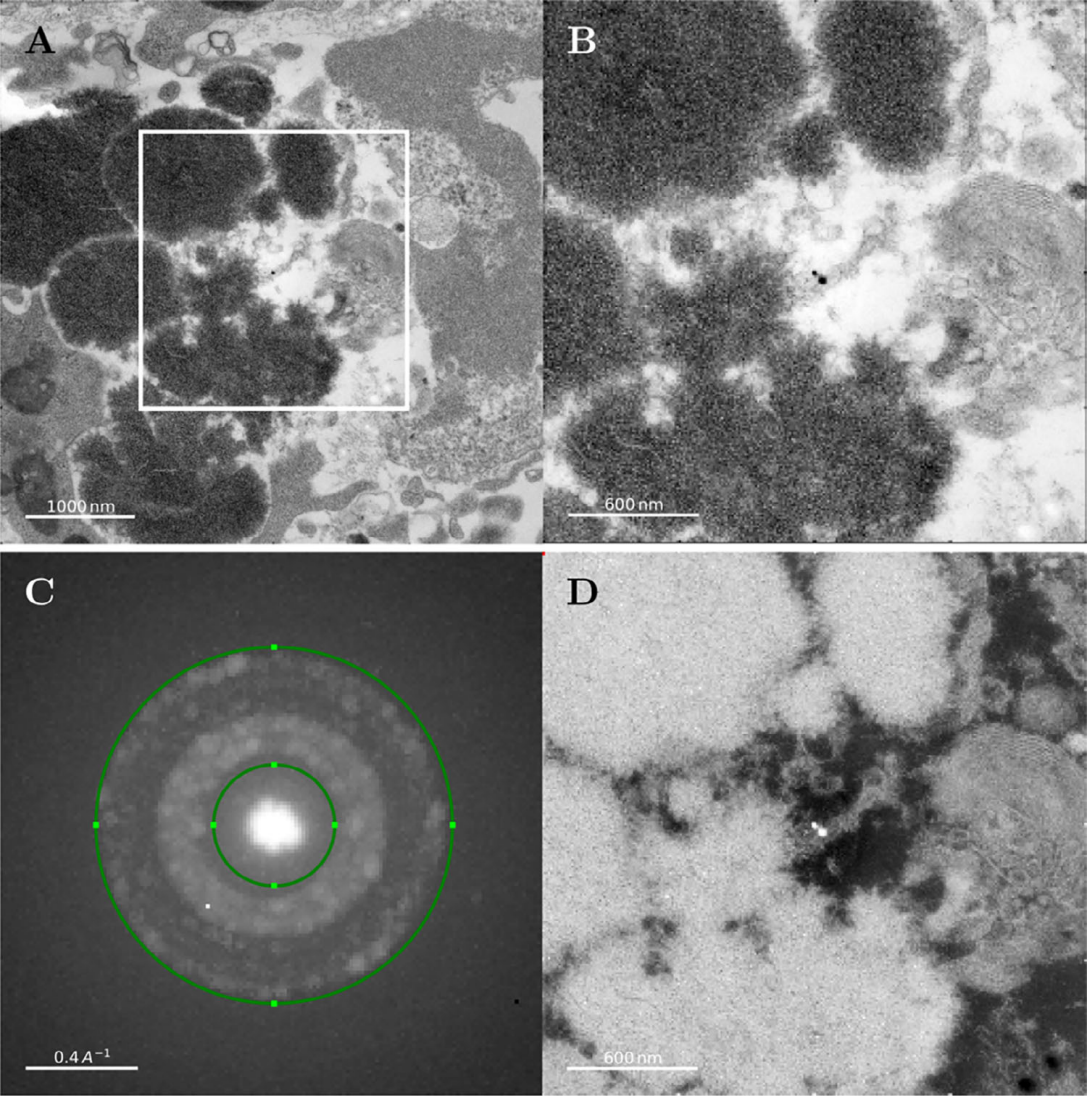
Scanning electron diffraction (SED) data for primary human osteoblasts (hOBs) spheroid transmission electron microscopy (TEM) sections at day 14 collected from an area similar to what is shown in Figure 3 (D14). In these experiments, electron diffraction data were collected for every pixel in the field of view. These data were then used to reconstruct bright‐field (transmission) and dark‐field micrographs. (*A*, *B*) Virtual bright‐field TEM images reconstructed from SED data. (*A*) Sample region that contains cellular structures (right) and large calcium phosphate (CaP) deposits (middle left, dark areas). (*B*) Magnified region from *A*. (*C*) Electron diffraction signal from the whole image shown in *B*. (*D*) Virtual annular dark‐field image reconstructed based on the diffraction signal from crystalline HAp confirming the presence of crystalline calcium phosphate in the area with the strong diffraction signal shown as bright areas in the reconstructed image.

We have used scanning electron diffraction technique to detect and investigate the distribution of crystalline HAp in samples cultured for 14 days. The same TEM sections used for standard TEM microscopy were examined using SED using a step size of 10 nm. A single electron diffraction pattern was collected for each probe position in the field of view (Fig. 4), and these diffraction patterns were used to reconstruct bright‐field and dark‐field images. An area similar to that shown in Figure 3 (day 14, region that includes mineral crystals) was examined. Virtual bright‐field TEM images (Fig. 3*A*, *B*) show dark crystalline regions with a morphology that resembles the morphology of the crystalline particles shown in Figure 3. Figure 4*C* shows accumulated electron diffraction signals from the whole image shown in Figure 4*B* and clearly indicates the presence of a crystalline phase. A virtual annular dark‐field image reconstructed using the diffraction signal range corresponding to the scattering angles of the HAp crystal structure is shown in Figure 4*D*. This image clearly highlights regions of the sample that contains crystalline HAp.

### Elemental analyses of the bonelike structure by SEM‐EDX

**Fig. 5:**
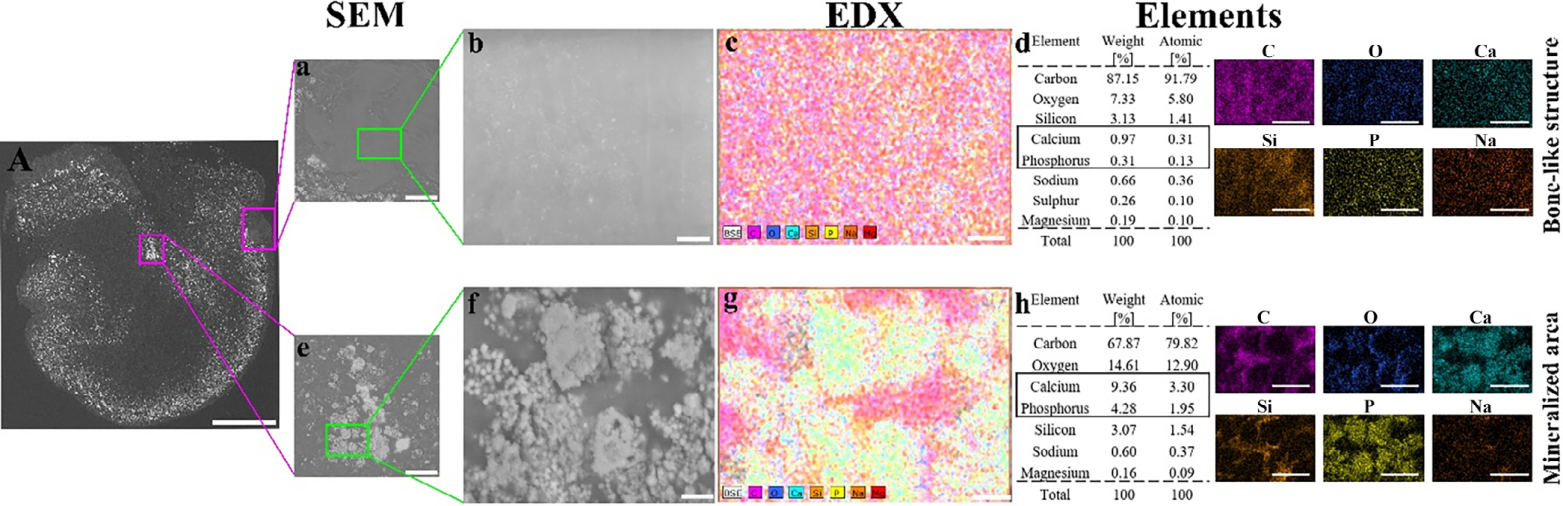
Morphology and elemental composition of an osteosphere at day 14. Scanning electron microscope (SEM) images showing the overall morphology of osteosphere (*A*) with the focus on bonelike structure and mineralized area marked in pink box. (*a*) The magnified area in the pink box shows the bonelike structure; (*e*) the magnified area in the pink box shows the mineralized area. The green box in (*a*) shows the bonelike structure and in (*e*) shows the mineralized areas of interest at high SEM signal density at higher magnification in (*b*) and (*f*) and were used in the energy‐dispersive X‐ray spectroscope (EDX) analyses. (*c*) The EDX maps of the selected area on bonelike structure in (*b*); (*g*) the EDX maps of the selected area on mineralized area in (*f*). (*d*) Element shows the elemental composition of the selected area in Fig. *c*‐EDX, and *h*‐Element shows the elemental composition of the selected area in Fig. *g*‐EDX. Scale bar = 500 μm (*A*), 100 μm (*a* and *e*), 30 μm (*b* and *f*), and 200 μm (*c*, *d*, *g*, and *h*).

The morphology and the quantitative elemental analysis of a section of an osteospheroid at D14 are shown in Figure 5. The mineral was identified mainly around the peripheral rim of the osteospheroids. Two different areas of an osteospheroid were selected for SEM and EDX analyses: Figure 5*a*, showing bonelike structure at higher magnification, and Figure 5*e*, showing mineralized area at higher magnification, marked in pink boxes in Figure 5*A*. Higher magnification of the bonelike structure (Fig. 5*b*) and EDX analysis (Fig. 5*c*) showed some minerals in areas with low SEM signal density. Atomic percentage (%) of calcium and phosphorous identified in the bonelike structure were 0.31 and 0.13, respectively (Fig. 5*d*). On the other hand, the SEM images of the area of high SEM signal density (Fig. 5*f*) and the following EDX analysis (Fig. 5*g*) of the mineralized area showed a different distribution of elements. Notably, the atomic % of calcium and phosphorous identified in the mineralized area was relatively high (3.30 and 1.95, respectively) (Fig. 5*h*), compared with the bonelike structure of low SEM signal density. The paraffin sections were mounted on glass slide for SEM‐EDX analysis. The EDX analysis of the pure glass slide showed only small amounts of Ca, but P was not detected (results not shown). Therefore, the atomic % of calcium identified in the osteospheroid may contain Ca signal originating from the glass slide on which the sample is mounted, but as the glass does not contain phosphorus, the detected P are from the osteospheroid only. In addition to that, SEM‐EDX analysis on the non‐visible collagenous and/or not mineralized area was used as a negative control (Supplemental Fig. S1).

### Cells entrapped in the bonelike structure

**Fig. 6:**
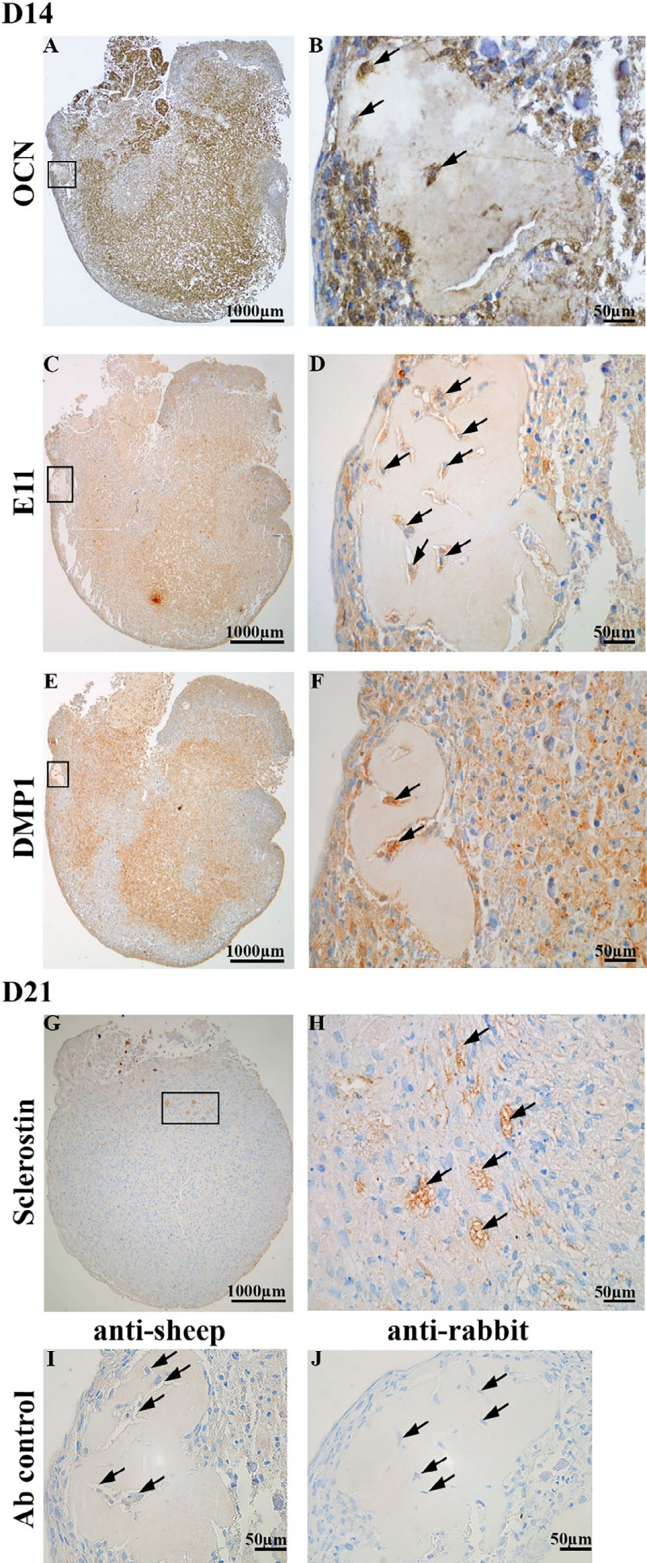
Light microscopy images of immunolabeling of the osteospheroid with OCN, E11, DMP1, and Sclerostin. Overview of the whole spheroid is shown in *A*, *C*, *E*, and *G*, whereas areas with a black square are shown with a higher magnification in *B*, *D*, *F*, and *H*. OCN (late osteoblast marker), E11 (early marker), and DMP1 (osteoid‐osteocyte marker) were expressed by the embedded osteocyte‐like cells on day 14 (D14) (*B*, *D*, *F*). Sclerostin (late marker) was expressed by a limited number of cells on day 21 (D21) (*G*, *H*). (*I*, *J*) The negative controls for the different secondary antibodies at D14.

The immunohistochemical analyses of the osteospheroids at D14 (Fig. 6) showed embedded cells in a bonelike structure with the late osteoblast (OCN), early osteocyte marker (E11), osteoid‐osteocyte marker (DMP1), and weak/no staining of late osteocyte marker (sclerostin) (data not shown). In addition, cells located within the central area of osteospheroid and within and close to loose collagen area (located on the top left side of spheroid section) were also observed to be positive for OCN, E11, and DMP1 staining (Fig. 6*A*, *C*, *E*). However, these cells were observed to be more strongly stained for OCN and DMP1 compared with E11. At D21, some cells located in a pericentral area of the osteospheroid were observed with strong sclerostin staining (Fig. 6*G*, *H*). On the other hand, staining of cryo‐embedded osteospheroid at D21 (Supplemental Fig. S2) with ALP reveal no or weak staining in general, whereas OCN staining showed no or weak staining of cells surrounded by dense collagen area (embedded cells) compared with cells surrounding the dense collagen area. E11 showed staining in the periphery area, whereas DMP1 and SOST showed the strongest signal from the cells in the center of the osteospheroid, indicating a differentiation to more mature osteocyte‐like cells (Supplemental Fig. S2). Sclerostin staining was observed to be more specific in the cells surrounded by dense collagen (Supplemental Fig. S2). These results taken together interpret a differentiation of the cells in the osteospheroid from osteoblasts toward osteocyte‐like cells within 14 to 21 days in the rotary culture.

## Discussion

The present study showed that human osteoblasts under scaffold‐free rotating culture conditions were able to form natural bonelike structures with differentiated osteocyte‐like cells embedded in the mineralized matrix produced by the cells. The cell phenotype was identified by the stellate appearance and cellular extensions originating from cell bodies and the expression of the late osteoblast and osteocyte markers, such as OCN, podoplanin/E11, DMP1, and sclerostin. The extracellular matrix (ECM) surrounding the cells had traces of mineral deposits and collagen with well‐defined banded fibrils, characteristic of type 1 collagen.

It has previously been shown that osteoblasts may differentiate into osteocytes in 3D scaffold culture systems in vitro; however, the complexity of in vivo tissue can only be replicated partially in such 3D models. Therefore, despite the increased feasibility of 3D in vitro models, an ideal in vitro model is yet to established.^(^ ^4^ ^)^ We have here verified that spheroids of osteoblasts, also referred to as osteospheroids, represent a new in vitro model for the development of osteocyte‐like cells. Slow rotation of cells in a vessel counter balanced the gravitational forces with velocity, lowered the shear forces, and promoted aggregation of the osteoblasts.^(^ ^14^ ^)^ Differentiation and maturation of the cells were promoted by the cellular density,^(^ ^37^ ^)^ cell‐to‐matrix contact,^(^ ^38^ ^)^ and continuous supply of nutrients, like vitamins, resulting in the formation of bone tissue‐like structures without the inclusion of any scaffold material. We have previously shown that primary human osteoblasts form a self‐assembled mineralized extracellular matrix within the 3D bone spheroids and that these spheroids represent a suitable model for the assessment of the effect of various stimuli on the biomechanical properties of bone.^(^ ^24^, ^25^ ^)^

In the present study, osteoblasts cultured in osteogenic medium with vitamins A, C, and D in a rotary culture for 14 to 21 days formed osteospheroids with a bonelike structure with embedded osteocyte‐like cells. These findings are reminiscent of in vivo osseous structures and are in agreement with the results of previous studies, showing the formation of bonelike nodules by culturing Wharton's jelly stem cells (WJ‐SCs) on calcium phosphate substrate^(^ ^39^ ^)^ and murine osteoblast cell line (MC3T3‐E1) and primary mouse osteoblast cell on type 1 collagen matrix.^(^ ^40^ ^)^ Efforts have been made to establish in vitro models for the study of osteocytes;^(^ ^39^, ^40^, ^41^, ^42^ ^)^ however, in these studies, osteoblasts were differentiated on collagen gels or calcium phosphate (CaP) scaffolds, which may interact with the osteocyte differentiation.^(^ ^4^ ^)^ In this study, we obtained cells embedded in the bonelike structure with a stellate appearance and cellular extensions extending from the cell body surrounded by a self‐excreted matrix, which is typical of osteocytes in their native environment. This supports a differentiation from osteoblasts to osteocyte‐like cells without the introduction of scaffolds. The extracellular matrix (ECM) in our study contained collagen and mineralized tissue. The collagen fibrils showed the characteristic appearance of well‐defined banded fibrils, which is a characteristic of type 1 collagen. These fibrils were observed throughout the osteospheroid, similar to the collagen fibrils reported in other studies where scaffolds have been applied.^(^ ^39^, ^40^, ^43^ ^)^

The mineralization process with entrapment of pre‐osteocytes plays an important role in the differentiation from pre‐osteocytes to late osteocytes.^(^ ^34^ ^)^ According to our TEM analysis, aggregates of needle‐shaped structures suggestive of crystals among collagen fibrils were identified at days 14 and 21 in the osteospheroids. In support of this, the presence of CaP was shown on day 14 by SEM‐EDX. These findings were similar to needlelike hydroxyapatite (HA) crystals among collagen fibrils identified in vitro and in a mice study.^(^ ^44^ ^)^ We could not identify the needle‐shaped aggregates in collagen‐rich bonelike areas in TEM image; however, SEM‐EDX showed the presence of Ca and P in low concentration, and SED showed the presence of crystalline Ca and P, in such areas that may indicate an early osteoid nature of these bonelike structures.^(^ ^14^, ^43^ ^)^

In addition to the morphological similarities to in vivo osteocytes in our study, these cells also expressed late osteoblast and osteocyte markers such as OCN, podoplanin/E11, DMP1, and sclerostin. OCN is a late‐stage marker expressed by mature osteoblast^(^ ^45^ ^)^ and is used as an indicator of osteogenic maturation.^(^ ^46^ ^)^ OCN in our study showed strong expression by entrapped cells on early time point (D14) and weak/no expression by cells surrounded by dense collagen on late time point (D21), which indicate the maturation of the osteoblast. E11, an early osteocyte marker, is expressed on the osteocyte surface and along the dendritic surface.^(^ ^47^ ^)^ The embedded osteocyte‐like cells in the bonelike structures in our study showed positive staining for E11, indicating osteoblast to osteocyte differentiation, which is in agreement with Keqin and colleagues,^(^ ^48^ ^)^ who confirmed the presence of E11 at osteoblast to osteocyte transition in different osteocyte cell lines.^(^ ^48^ ^)^ DMP1 is mainly expressed in the stage of late osteoblast to osteocyte (osteoid‐osteocyte) development^(^ ^49^ ^)^ and control osteocyte maturation.^(^ ^50^ ^)^ In our study, DMP1 was shown in the osteocyte‐like cells on day 14. This suggests that cells entrapped in bonelike structure in our study are more inclined to have osteocyte phenotype because the expression of DMP‐1 is far higher in osteocytes^(^ ^51^ ^)^ and very weak in osteoblasts. In addition, it seems like the C‐terminal end of the DMP1 is the active part mostly expressed in osteocytes,^(^ ^52^ ^)^ which corresponds to the antibody used in our study, which further validates DMP1 protein expression in our results on day 14. Sclerostin is identified as a late osteocyte marker and is expressed in mature and deeply embedded osteocytes.^(^ ^53^ ^)^ This may explain why sclerostin in our study was weakly expressed in the osteocyte‐like cells on day 14, and on day 21 strong expression was only observed in a limited number of cells. Our results are in agreement with Sun and colleagues,^(^ ^12^ ^)^ who also showed that expression of sclerostin increased with longer culture time of human osteoblast.^(^ ^12^ ^)^ However, we do lack the staining of specific osteoblast markers, but as our model only contained commercial primary human osteoblast cells, we assumed that cells stained either positively or negatively with osteocytic marker may indicate different stages of osteoblast to osteocyte differentiation. Hence, our model may indicate a continuous maturation within the osteospheroid of the bonelike structures and the cells.

Vitamins are essential for bone formation, and vitamins A, C, and D play important roles in the osteoblast to osteocyte differentiation. However, only a handful of studies have been performed on some combination of these vitamins to study the antagonist effect^(^ ^54^ ^)^ or effect on bone mineral density and fragility.^(^ ^55^, ^56^ ^)^ Vitamin supplementation in rotary culture seemed to favor the transition from osteoblasts to osteocyte‐like cells, which is in agreement with other studies where vitamin A supplementation enhanced osteoblast to osteocyte differentiation,^(^ ^57^, ^58^ ^)^ vitamin C increased osteoblast differentiation, mineralization,^(^ ^59^ ^)^ extracellular matrix (ECM) synthesis, and regulated ECM/collagen homeostasis,^(^ ^60^ ^)^ and vitamin D supplementation promoted differentiation and matrix mineralization.^(^ ^61^, ^62^ ^)^ In the present study, the cocktail of vitamins was given continuously and in the same concentration throughout the experiment. To identify the cellular mechanisms of vitamins, alone and in combination, in our experimental setup, more studies are needed. Additional exploration of these osteospheroids is needed, including ECM component surrounding the osteocyte‐like cells, osteocyte‐like cell morphology and signaling mechanism, and sensing of the dynamic environment by osteocyte‐like cells. Furthermore, osteospheroids generated using BAM and vitamins could be used as a tool to explore the communication mechanism of osteocyte‐like cells in its de novo microenvironment. Taken together, the creation of osteospheroids with the presence of osteoblasts and osteocyte‐like cells embedded in osteoid/bonelike structure may be a promising tool in exploring the functions of the osteocytes and communication of osteocytes with surrounding environment. Moreover, factors involved in the transition of osteoblasts to osteocytes may be studied in this natural scaffold‐free environment.

The present study shows that human osteoblasts in a scaffold‐free rotary culture condition can create a natural bonelike structure with osteocyte‐like cells embedded in a secreted, mineralized collagen matrix. Our system may be a promising tool to study osteoblast to osteocyte transition, osteocyte communication, and the responses to various agents.

## Author Contributions

**Arooj Munir:** Data curation; formal analysis; investigation; methodology; validation; visualization; writing – original draft; writing – review and editing. **Janne Elin Reseland:** Conceptualization; formal analysis; funding acquisition; methodology; project administration; supervision; validation; writing – original draft; writing – review and editing. **Hanna Tiainen:** Data curation; methodology; validation; writing – review and editing. **Håvard Jostein Haugen:** Methodology; validation; writing – review and editing. **Pawel Sikorski:** Data curation; formal analysis; methodology; validation; visualization; writing – review and editing. **Emil Frang Christiansen:** Data curation; formal analysis; methodology; validation; visualization; writing – review and editing. **Finn Per Reinholt:** Funding acquisition; validation; writing – review and editing. **Unni Syversen:** Conceptualization; validation; writing – review and editing. **Lene Bergendal Solberg:** Conceptualization; data curation; formal analysis; funding acquisition; investigation; project administration; resources; supervision; validation; visualization; writing – original draft; writing – review and editing.

## Disclosures

The authors declare no conflicts of interest.

### Peer Review

The peer review history for this article is available at https://www.webofscience.com/api/gateway/wos/peer-review/10.1002/jbm4.10792.

## Supporting information


**Data S1.** Supporting Information
