# Supplementary material for: Osteocyte‐Like Cells Differentiated From Primary Osteoblasts in an Artificial Human Bone Tissue Model
Source: JBMR Plus. 2023 Jun 28;7(9):e10792. doi: 10.1002/jbm4.10792 (PMC10494512; doi:10.1002/jbm4.10792)
Supplement: Supplementary file 1 — Data S1. Supporting Information [file JBM4-7-e10792-s001.docx]

**Osteocyte-like cells differentiated from primary osteoblasts in an artificial human bone tissue model**

Arooj Munir^a^, Janne E. Reseland^a^, Hanna Tiainen^a^, Håvard J. Haugen^a^, Pawel Sikorski^b^, Emil Frang Christiansen^b^, Finn P. Reinholt^c^, Unni Syversen^d^, Lene B**.** Solberg^c,e *^

^a^Department of Biomaterials, Institute of Clinical Dentistry, University of Oslo, Oslo, Norway; [arooj.munir@odont.uio.no](mailto:arooj.munir@odont.uio.no), [j.e.reseland@odont.uio.no](mailto:j.e.reseland@odont.uio.no), [hanna.tiainen@odont.uio.no](mailto:hanna.tiainen@odont.uio.no), [h.j.haugen@odont.uio.no](mailto:h.j.haugen@odont.uio.no)

^b^Department of Physics, Norwegian University of Science and Technology (NTNU), Trondheim, Norway; [pawel.sikorski@ntnu.no](mailto:pawel.sikorski@ntnu.no), [emil.christiansen@ntnu.no](mailto:emil.christiansen@ntnu.no)

^d^Department of Clinical and Molecular Medicine, Norwegian University of Science and Technology, Trondheim, Norway; [unni.syversen@ntnu.no](mailto:unni.syversen@ntnu.no)

^c^Department of Pathology and ^e^Division of Orthopaedic Surgery, Oslo University Hospital, Oslo, Norway; [Finn.P.Reinholt@rr-research.no](mailto:Finn.P.Reinholt@rr-research.no), [l.b.solberg@gmail.com](mailto:l.b.solberg@gmail.com)

*** Corresponding author**

Lene Bergendal Solberg MD, PhD

Department of Pathology and Division of Orthopaedic Surgery

Oslo University Hospital

Postbox 4950 Nydalen, N-0424 OSLO, Norway

Phone: +47 22 11 80 80

E-mail address: [l.b.solberg@gmail.com](mailto:l.b.solberg@gmail.com) (L.B.Solberg)

**Supplementary Figure 1**


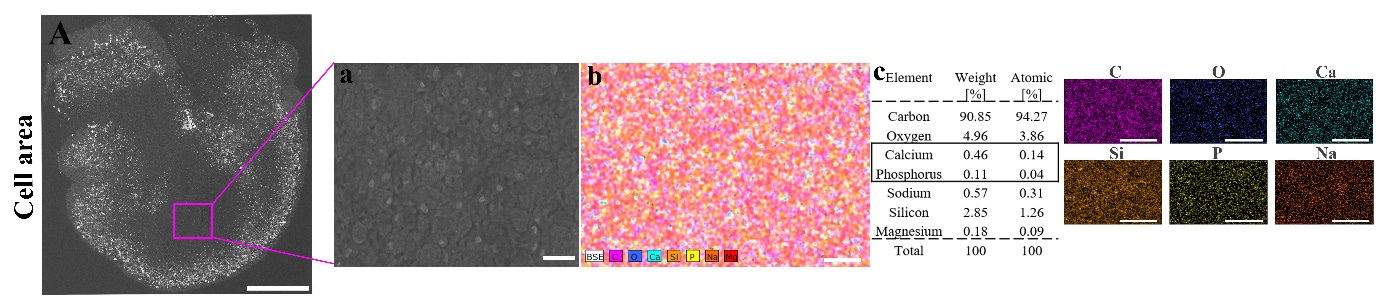


**Supplementary Figure 1.** Morphology and elemental composition of osteosphere (**A**) at D14, with the focus on cell area in pink box. **a** shows the magnified area in pink box showing cells in osteosphere. **b** shows the cell area of interest at high SEM signals density and **c** shows the elemental composition of the selected area in **b**. Scale bar 500 µm (**A**), 30 µm (**b**) and 200 µm (**c**).

**Supplementary Figure 2**


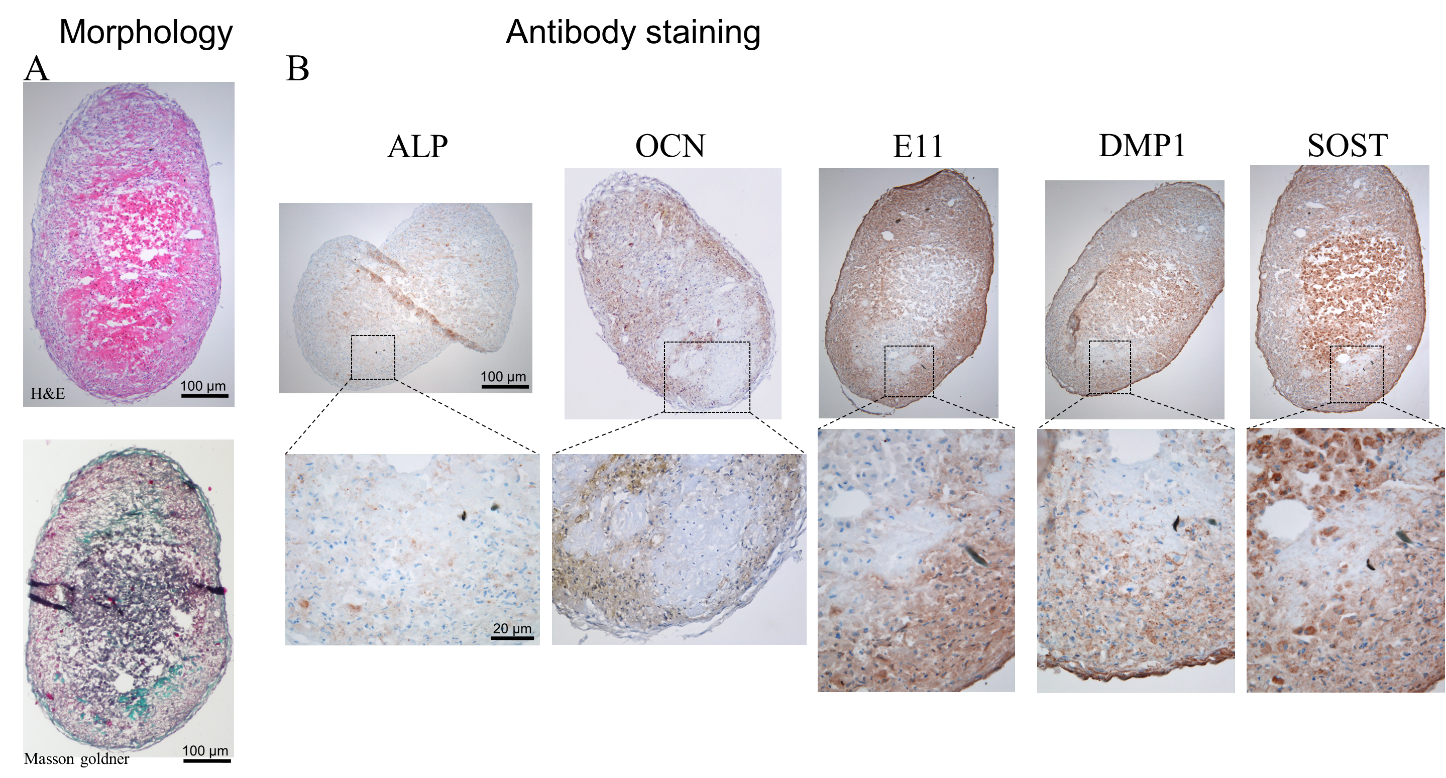


**Supplementary Figure 2**. Osteospheroid embedded in sucrose and liquid nitrogen for cryo-sectioning of the ostespheroid at D21. **A** shows the areas of dense collagen in the lower middle of the spheroid and close to the boarder. **B** shows immunohistological staining of the osteospheroid. ALP only reveal weak or no signal from the cells in general; OCN reveals weak or no signals of cells surrounded by dense collagen (osteocyte-like cells) area compared to the rest of the cells, while E11, DMP1 and SOST reveal strong cell signals. E11 (an early osteocyte marker) demonstrated staining in the periphery while DMP1 and SOST showed the strongest signal from the cells in the center of the osteospheroid indicating a differentiation to more mature osteocytes.

**Supplementary Figure 2. Materials and methods**

**Histological tissue preparation**

3D spheroids were obtained by following the cell expansion, and 3D cell culture method as mentioned the materials and methods section. The spheroid harvested on D21 was washed with PBS, fixed with 4% PFA paraformaldehyde for 15 minutes, and embedded in optimal cutting temperature (OCT) frozen sectioning medium (VWR International BVBA, Leuven, Belgium). Sections of 10 μm thickness were obtained using a CryoStar NX70 cryostat (Thermo Fisher Scientific, Waltham, MA, USA) and mounted on glass slide and stored at -20°C.

*Masson-Goldner staining*

Nuclei were stained for 5 minutes with Weigart’s haematoxylin (Chemi-teknik AS, Oslo, Norway), followed by rinsing with acid alcohol and water. Subsequently, the tissue components were stained with ponceau-syrefuchsin-azophloxin (Fuchsin acid: MERCK, Darmstadt, Germany; Ponceau: Chemi-teknik AS, Oslo, Norway) for 7 minutes, orange G (Sigma-Aldrich) for 5 minutes, and 0.5% light green (Chemi-teknik AS, Oslo, Norway) for 7 minutes, and in between washing with 1% acetic acid was done. Finally, sections were rinsed in acetic acid, dehydrated quickly through graded ethanol, and xylene and mounted. The mounted sections were analyzed using light microscopy (Olympus BX51 microscope (Tokyo, Japan)).

*Immunohistochemistry*

Antigen retrieval was carried out by incubating the sections with hyaluronidase (15.000 U/ml, Sigma Aldrich) in a hot cabinet at 37^°^C for 30 minutes, followed by EnVision FLEX Wash Buffer 1x (Dako-Agilent, Santa Clara, US). Sections were blocked with endogenous peroxidase (Dako-Agilent) for 5 minutes and 1% BSA (Sigma-Aldrich) for 30 minutes, both at room temperature (RT), before incubation with the respective primary antibodies: rabbit DMP1 (1:200; HPA037465; Sigma-Aldrich), rabbit Sclerostin (1:50; ab85799; Abcam, Cambridge, UK), sheep Podoplanin (1:50; AF3670; R&D Systems, Minneapolis, USA), mouse alkaline phosphatase ALPL (1:20; MAB1448; R&D Systems), and mouse OCN (1:100; 33-5400; Thermo Fisher Scientific, Inc.) overnight at 4°C. Incubation with appropriate secondary antibodies envision + system-HRP anti rabbit, anti mouse (Dako-Agilent), and IgG HRP-conjugated anti sheep (1:500; R&D systems) were performed for 30 minutes at RT followed by washing buffer. Finally, the sections were stained with hematoxylin, dehydrated, and mounted. The mounted sections were analyzed using an Olympus BX51 microscope.
